# Clinical Characteristics, Empirical Antibiotic Appropriateness, and Outcomes of Gram-Positive Versus Gram-Negative Bacteremia in Emergency Department Patients with Sepsis

**DOI:** 10.3390/medicina62071353

**Published:** 2026-07-13

**Authors:** Taekwon Kim, Jonghoon Yoo, Sang-hun Lee, Wondong Jung

**Affiliations:** 1 Department of Emergency Medicine, Keimyung University School of Medicine, Keimyung University Dongsan Hospital, Daegu 42601, Republic of Korea; wsnatz@gmail.com (J.Y.); sanghun@dsmc.or.kr (S.-h.L.); 2 Department of Emergency Medicine, Keimyung University Dongsan Hospital, Daegu 42601, Republic of Korea; jwd7357@gmail.com

**Keywords:** sepsis, bacteremia, Gram-positive bacteremia, Gram-negative bacteremia, emergency department, empirical antibiotic therapy, mortality

## Abstract

*Background and Objectives*: Bacteremia is associated with worse outcomes in sepsis, but comparative data between Gram-positive bacteremia (GPB) and Gram-negative bacteremia (GNB) remain scarce. We compared clinical characteristics, empirical antibiotic appropriateness, and outcomes of GPB and GNB in emergency department (ED) patients with sepsis. *Materials and Methods*: We retrospectively studied ED patients who met the Sepsis-3 criteria with culture-confirmed GPB or GNB from December 2021 to August 2024. The primary outcome was 28-day mortality. Secondary outcomes included 90-day mortality, in-hospital mortality, ICU admission, and length of hospital stay. Multivariable logistic regression assessed the association between Gram status and 28-day mortality. *Results*: Of 493 patients, 96 had GPB and 397 had GNB. GPB was associated with higher 28-day mortality (26.0% vs. 9.3%), 90-day mortality (34.4% vs. 11.8%), in-hospital mortality (26.0% vs. 10.1%), ICU admission (36.5% vs. 24.7%), longer hospital stay (20.5 [11.8–31.0] vs. 14.0 [9.0–20.0] days), and substantially more frequent inappropriate empirical antibiotic therapy (37.5% vs. 12.8%) (all *p* < 0.05). The independent association between GPB and 28-day mortality was not consistent across adjusted models and was attenuated after accounting for inappropriate empirical antibiotic therapy (aOR, 1.85; 95% CI, 0.92–3.71). In exploratory analyses, GPB was associated with four-fold higher odds of inappropriate empirical antibiotic therapy than GNB (aOR, 4.07; 95% CI, 2.19–7.54). *Conclusions*: In bacteremic sepsis, GPB was associated with worse observed outcomes and substantially more frequent inappropriate empirical antibiotic therapy than GNB. GPB should be interpreted less as a standalone microbiologic determinant of mortality and more as a marker of a clinically vulnerable and management-challenging profile. Our findings point to a modifiable diagnostic–treatment gap in early ED recognition and empirical antibiotic selection. Prospective multicenter studies should evaluate whether systematic reassessment of clinical features suggestive of GPB and suspected infection source can improve empirical antibiotic appropriateness and ultimately translate into better clinical outcomes.

## 1. Introduction

Sepsis is life-threatening organ dysfunction caused by a dysregulated host response to infection [1]. Each year, it affects roughly 49 million people and causes about 11 million deaths, representing nearly 20% of global mortality [2]. The burden of this condition extends to health systems, which expend substantial resources on care and bear rising costs [2]. Bacteremia denotes the presence of viable microorganisms in normally sterile blood and may occur either as a primary event or secondarily through dissemination from a localized infection site. Bacteremia does not always produce sepsis, and sepsis often arises without it. When the two coincide, however, illness severity escalates and prognosis worsens [3,4,5].

Recent epidemiologic studies report a rising proportion of Gram-positive organisms among bacteria causing bloodstream infections and sepsis, partly because of the wider use of invasive medical devices and the increasing prevalence of antibiotic-resistant Gram-positive pathogens [6,7,8]. This trend bears directly on emergency department (ED) care, where clinicians must initiate resuscitation, evaluate the suspected source of infection, and select empirical antibiotics before culture identification and susceptibility results are available. Gram-positive and Gram-negative organisms differ in cell-envelope structure and in certain drug targets, which together influence the range of effective antimicrobial agents [9,10]. Because the causative organism and its susceptibility profile cannot be confirmed at the time of empirical selection, early therapy depends instead on recognizing clinical features suggestive of Gram-positive organisms, such as healthcare exposure, hemodialysis or vascular access, indwelling devices, and suspected endovascular or device-associated infection. This distinction matters because standard empirical regimens for suspected sepsis often cover Gram-negative organisms, whereas Gram-positive coverage is typically added selectively according to suspected infection source, host risk factors, prior microbiology, and local resistance patterns [1]. Because early appropriate antimicrobial therapy and timely source control are among the strongest determinants of survival in sepsis [11,12,13,14,15], identifying the likely source, anticipating Gram-positive bacteremia (GPB) or Gram-negative bacteremia (GNB), and selecting appropriate empirical therapy at presentation are central to early ED diagnosis and treatment. When GPB is not anticipated, this early opportunity can be missed, with potential consequences for early risk stratification and short-term mortality in bacteremic sepsis.

Previous studies have compared sepsis caused by Gram-positive and Gram-negative organisms, but very few studies have specifically considered patients with culture-proven bacteremia. Prognostic findings have also been inconsistent, with some reporting worse outcomes with Gram-positive infections, others reporting worse outcomes with Gram-negative infections, and still others finding no clear difference [16,17,18,19]. Variation in patient selection, the inclusion of non-bacteremic infections, and incomplete adjustment for comorbidity burden, illness severity, infection source distribution, and empirical antibiotic appropriateness may have contributed to this inconsistency. Because culture-proven bacteremia represents a clinically high-risk subgroup within sepsis, GPB and GNB warrant direct comparison in this population.

We therefore compared the clinical characteristics, empirical antibiotic appropriateness, and clinical outcomes of GPB and GNB in adult patients with sepsis presenting to the ED. We also evaluated whether Gram status was independently associated with 28-day mortality beyond differences in comorbidity burden, illness severity, infection source, and the appropriateness of empirical antibiotic therapy. Through this approach, we aimed to clarify whether outcome differences between GPB and GNB reflect Gram status itself or are better explained by accompanying patient-, source-, and treatment-related factors.

## 2. Materials and Methods

### 2.1. Study Design and Population

We conducted this retrospective observational cohort study at the ED of a single tertiary university hospital from December 2021 to August 2024. Sepsis patients were classified as having either GPB or GNB based on initial blood cultures at ED presentation, then compared for clinical characteristics, empirical antibiotic appropriateness, and outcomes. The clinical variables collected a priori included functional status, place of acquisition, comorbidities, central venous catheter use, the Charlson Comorbidity Index, multidrug-resistant (MDR) organism status, infection source, septic shock, the Sequential Organ Failure Assessment (SOFA) score, the Acute Physiology and Chronic Health Evaluation II (APACHE II) score, initial vital signs, and early organ-support therapies. Comorbidities comprised hypertension (HTN), diabetes mellitus (DM), heart failure, chronic lung disease, non-dialysis chronic kidney disease (CKD), end-stage renal disease (ESRD) on hemodialysis, chronic liver disease, solid-organ transplantation, solid tumor, and hematologic malignancy. Hematologic malignancy was recorded according to the operational definition provided in Section 2.3. Immunocompromise-related variables, including severe neutropenia, recent chemotherapy, and systemic corticosteroid therapy, were recorded for all patients. To avoid inferring marrow dysfunction from the underlying hematologic diagnosis alone, marrow function at ED presentation was assessed objectively using the absolute neutrophil count.

We screened adult patients (≥18 years) presenting to the ED by applying diagnostic codes for sepsis, septic shock, or infection-related diagnoses based on the Korean Standard Classification of Diseases, 8th Revision (KCD-8), the Korean national disease classification system based on the International Classification of Diseases, 10th Revision (ICD-10). We applied these codes to generate an electronic screening list of potentially eligible adult ED patients. Four trained investigators then performed manual chart review of every screened case to confirm suspected or proven infection, Sepsis-3 eligibility, ED blood culture positivity, final organism identification, infection source, exclusion criteria, and outcomes. Cases with uncertain eligibility, source attribution, or contaminant status were adjudicated by consensus with a senior investigator. Diagnostic codes were therefore used only for screening, whereas final inclusion required chart-confirmed infection-associated organ dysfunction and culture-confirmed monomicrobial bacteremia. The KCD-8 code list used for the electronic screen is provided in Supplementary Table S1.

Sepsis followed the Sepsis-3 criteria and was defined as infection-associated organ dysfunction, operationalized as an acute increase of at least 2 points in the SOFA score. When pre-existing organ function data were available, organ dysfunction was defined as an acute increase of ≥2 points in the SOFA score from baseline. For each patient, SOFA component variables were extracted electronically at two time points, the most recent stable pre-illness period before the index ED visit and the first 24 h after ED arrival, and were then reviewed to estimate baseline component scores and to identify the worst eligible values within this 24 h window. For patients with documented chronic renal, hepatic, respiratory, neurologic, hematologic, or cardiovascular dysfunction, chronic abnormalities were not counted as acute sepsis-related organ dysfunction, and acute deterioration was determined by comparison with pre-illness baseline values. When baseline values were unavailable or insufficient, acute deterioration was adjudicated using predefined component-specific criteria. For the neurologic, respiratory, and cardiovascular components, findings at presentation such as altered mental status, respiratory compromise, hypotension, or a vasopressor requirement were counted as acute only when history taking or chart review supported a new change from the patient’s pre-illness status. When the initial PaO_2_/FiO_2_ ratio could not be calculated, the respiratory component of the SOFA score was adjudicated as 0 points only when chart review confirmed that the patient was on room air with an SpO_2_ of 99% to 100% and had no clinical evidence of acute respiratory dysfunction during the first 24 h. For the hepatic, renal, and hematologic components, abnormal values at presentation were counted as acute only when repeat testing within 24 h showed further deterioration. Two renal situations were handled specifically: in patients receiving maintenance hemodialysis, chronically elevated creatinine was not counted as acute renal dysfunction, and in patients with non-dialysis CKD and no available baseline, acute renal dysfunction was counted only when new oliguria or anuria, progressive worsening on repeat testing within 24 h, or a new requirement for renal replacement therapy was documented. After these rules were applied, Sepsis-3 eligibility was determined by an acute SOFA increase of at least 2 points from baseline, or, when baseline data were unavailable, by a SOFA score of at least 2 calculated from the worst eligible component values recorded within 24 h of ED arrival.

**Figure A1:**
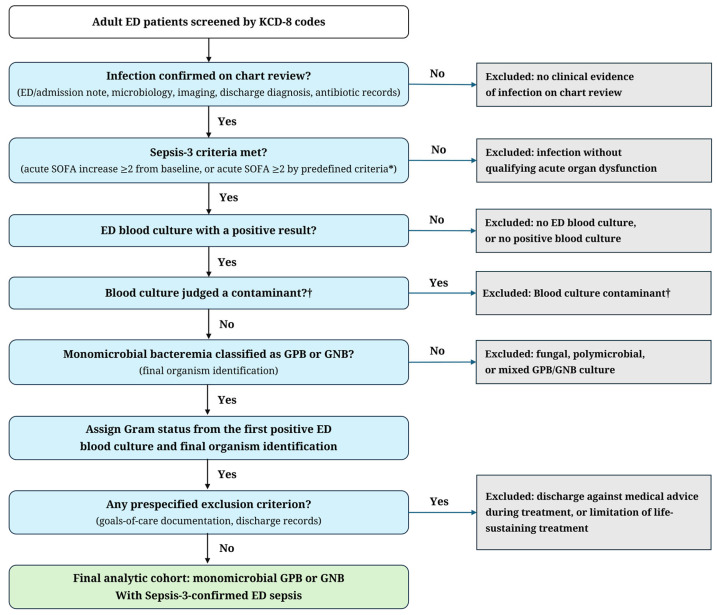
Cohort assembly and chart-review algorithm. Adult ED patients were initially screened using the KCD-8 codes listed in Supplementary Table S1 and then assessed by sequential manual chart review to confirm infection, fulfillment of the Sepsis-3 criteria, a positive ED blood culture that was not judged to represent contamination, and monomicrobial Gram-positive or Gram-negative bacteremia before inclusion in the final analytic cohort. * When baseline organ function data were unavailable or insufficient, or when an abnormality was chronic, acute organ dysfunction was adjudicated using predefined component-specific criteria and scored from the worst eligible component values within 24 h of ED arrival. For the neurologic, respiratory, and cardiovascular components, abnormalities at presentation were counted as acute only when history or chart review supported a new change from the patient’s pre-illness status. For the hepatic, renal, and hematologic components, abnormal values were counted as acute only when repeat testing within 24 h showed further deterioration, and for the renal component in patients with non-dialysis CKD and no available baseline, also when new oliguria or anuria or a new requirement for RRT was documented. Chronic abnormalities, including maintenance hemodialysis, were not counted as acute organ dysfunction. † Blood cultures were adjudicated for contamination on combined clinical and microbiologic review. For common skin commensals, including coagulase-negative staphylococci, *Corynebacterium*, *Bacillus* other than *B. anthracis*, *Cutibacterium*, and *Micrococcus* species, growth of the same organism in at least two separate sets was regarded as strong evidence of true bacteremia. A single-set commensal isolate was retained as true bacteremia only when supported by repeat positivity, a compatible clinical course, an identifiable source such as catheter-related infection, infective endocarditis, or prosthetic material infection, or documented targeted treatment. Single-set commensals without such support, or judged contaminants on clinical review, were excluded. Recognized non-skin pathogens, including *Staphylococcus aureus*, *Streptococcus pneumoniae*, beta-hemolytic streptococci, *Enterococcus* species, Enterobacterales, and *Pseudomonas aeruginosa*, were not classified as contaminants on the basis of a single positive set alone. Collection technique is detailed in the Blood culture collection paragraph above. ED, emergency department; GNB, Gram-negative bacteremia; GPB, Gram-positive bacteremia; KCD-8, Korean Standard Classification of Diseases, 8th Revision; SOFA, Sequential Organ Failure Assessment; CKD, chronic kidney disease; RRT, renal replacement therapy.

Among patients meeting these criteria, we included those with monomicrobial GPB or GNB identified from blood cultures obtained in the ED before antibiotic administration. Under the institutional ED sepsis protocol, blood cultures are drawn at presentation whenever bloodstream infection or sepsis is suspected, particularly with systemic or unstable features such as fever, hypotension, hypoxemia, or altered mental status not attributable to stroke. When cultures were not obtained during the initial evaluation but therapeutic antibiotics were subsequently indicated for a suspected infection, as distinct from surgical or procedural prophylaxis, cultures were drawn before the first antibiotic dose. The index cultures used for Gram-status classification were therefore obtained before antibiotics in all included patients. At least two sets were collected from separate peripheral venipuncture sites whenever feasible, and the first positive ED culture defined Gram status. When a common skin commensal grew in only one of these sets, two further sets were obtained from a fresh peripheral venipuncture site to distinguish true bacteremia from contamination. Repeat positive cultures, when available, were reviewed to support true bacteremia, source attribution, and contaminant adjudication. Common skin commensals, including coagulase-negative staphylococci, were classified as true pathogens only when corroborated by repeat positive cultures, a compatible clinical course, an identifiable source such as catheter-related infection or infective endocarditis, or documented targeted treatment, whereas recognized non-skin pathogens such as *S. aureus*, Enterobacterales, and *P. aeruginosa* were not classified as contaminants on the basis of a single positive set alone. Figure A1 (Appendix A) summarizes the complete cohort assembly and chart-review algorithm, including KCD-8 code-based screening, Sepsis-3 confirmation, ED blood culture filters, contaminant adjudication, Gram-status assignment, and prespecified exclusions.

The exclusion criteria were as follows: age < 18 years; blood culture isolates judged to be contaminants on clinical and microbiological grounds; fungal bloodstream infections; mixed Gram-positive and Gram-negative bacteremia; limitation of life-sustaining treatment, defined as a documented decision to withhold or withdraw vasopressors, mechanical ventilation, or renal replacement therapy; and discharge against medical advice during treatment. Patients with limitation of life-sustaining treatment were excluded because mortality in this group may be driven primarily by decisions to limit organ-supportive treatment rather than by infection severity or the appropriateness of empirical therapy.

### 2.2. Data Collection

From the electronic medical records, we collected data on demographics (age, gender, BMI), comorbid conditions, functional status within one week before presentation, place of acquisition (community-acquired, nosocomial, or nursing home-acquired), source of infection, causative pathogens and their antimicrobial resistance profiles, vital signs at presentation (blood pressure, heart rate, respiratory rate, body temperature), arterial blood gas (ABG) analysis and lactate at presentation, laboratory findings at presentation (white blood cell [WBC], neutrophil-to-lymphocyte ratio [NLR], hemoglobin, platelet, blood urea nitrogen [BUN], creatinine, sodium, albumin, and C-reactive protein [CRP], procalcitonin).

We also recorded the occurrence of septic shock, the initial fluid resuscitation volume, the time to empirical antibiotic administration, the appropriateness of empirical antibiotic therapy, the use of mechanical ventilation and renal replacement therapy during the first 24 h, use of vasopressors, corticosteroid use for the management of septic shock, and clinical outcomes, including 28-day mortality, 90-day mortality, in-hospital mortality, ICU admission, and length of hospital stay. The Charlson Comorbidity Index, the APACHE II score, and the SOFA score were calculated based on the collected clinical and laboratory data. We confirmed survival at 28 and 90 days from outpatient medical records, conducting telephone follow-up when this could not be established from the records.

### 2.3. Definitions

Inappropriate empirical antibiotic therapy was defined as either the failure to administer at least one systemic antimicrobial agent with in vitro activity against the isolated pathogen within 24 h of ED presentation, or the administration of a regimen clinically discordant with established guidelines for the identified pathogen or infection site [20,21,22,23,24,25,26,27]. The 24 h window defined the early empirical phase and was not meant to allow delayed treatment. It differs from the guideline focus on how quickly the first dose is given, because appropriateness depends instead on whether the chosen antibiotics are active against the pathogen that later grows. This window covered the initial ED regimen and any change made on the same day, before culture and susceptibility results were available. This approach matches prior bloodstream infection studies, in which appropriate antibiotic therapy given within 24 to 48 h of blood culture predicted mortality, and inappropriate antibiotic therapy raised mortality from about 12 h onward [28,29,30,31]. Two blood culture sets were drawn from separate peripheral sites at presentation, before antibiotics, in all included patients, and three sets were drawn when infective endocarditis was suspected. ED arrival and blood culture collection were therefore nearly simultaneous in this cohort. Repeat positivity, the clinical course, and an identifiable source distinguished true bacteremia from contamination. The full collection and adjudication protocol appears in Appendix A. Appropriateness was retrospectively assessed once final blood culture identification and susceptibility results were available. Examples of guideline-discordant or inappropriate regimens included third-generation cephalosporins for *Enterococcus* infections, suboptimal therapy for methicillin-susceptible *Staphylococcus aureus* (MSSA) infective endocarditis, daptomycin for pneumonia, and nitrofurantoin or fosfomycin for pyelonephritis or complicated urinary tract infection. Two investigators independently evaluated empirical antibiotic appropriateness, and disagreements were resolved by consensus with a senior investigator. Inter-rater agreement prior to consensus was assessed using Cohen’s kappa (κ) coefficient.

Hematologic malignancy was defined as leukemia, myelodysplastic syndrome, myeloproliferative neoplasm, lymphoma, multiple myeloma, or another plasma-cell or hematolymphoid malignancy, and was restricted to patients receiving disease-directed treatment at ED presentation. Remote disease in remission without current disease-directed treatment was not classified as hematologic malignancy. Severe neutropenia was defined as an absolute neutrophil count (ANC) below 500 cells/µL on the first complete blood count with differential obtained at ED arrival. Recent chemotherapy was defined as cytotoxic chemotherapy or disease-directed systemic anticancer therapy within the preceding 3 months. Systemic corticosteroid therapy was defined as more than 20 mg of prednisolone per day, or an equivalent dose, for at least 2 weeks within the preceding month.

Functional status in the week before presentation was classified as independent, partially dependent, or dependent according to the need for assistance with activities of daily living. Place of acquisition was classified as community-acquired when the infection was present at ED arrival in a patient coming from the community, nosocomial when onset occurred more than 48 h after admission or in relation to recent healthcare contact, and nursing home-acquired when the patient came from a nursing home or long-term care facility. The source of infection was assigned by the treating team and verified on chart review using clinical, microbiologic, and imaging data. Septic shock followed the Sepsis-3 definition, requiring vasopressors to maintain a mean arterial pressure of at least 65 mmHg together with a serum lactate above 2 mmol/L despite adequate fluid resuscitation [1]. MDR bacteria were defined as isolates non-susceptible to at least one agent in three or more antimicrobial categories. Early organ-support therapies comprised mechanical ventilation, renal replacement therapy, and vasopressor use initiated within 24 h of ED arrival.

### 2.4. Sepsis Management

Sepsis management followed Surviving Sepsis Campaign (SSC) guidelines and included early fluid resuscitation with crystalloids and prompt empirical antibiotics after blood cultures were obtained [1]. Empirical antibiotics were given at guideline-recommended doses and adjusted for renal function. In patients with septic shock, vasopressors, corticosteroids, mechanical ventilation, renal replacement therapy, and other supportive care were provided as clinically indicated.

### 2.5. Outcomes

The primary outcome was 28-day mortality. We also assessed 90-day mortality, in-hospital mortality, ICU admission, and length of hospital stay as secondary outcomes.

### 2.6. Statistical Analysis

We tested continuous variables for normality with the Shapiro–Wilk test and reported them as the mean ± standard deviation when normally distributed or as the median (interquartile range) when not. Groups were compared with Student’s *t*-test or the Mann–Whitney U test, as appropriate. Categorical variables were presented as numbers (%) and were compared with the chi-square test or Fisher’s exact test.

Univariable logistic regression was performed to evaluate the unadjusted association between each candidate variable and 28-day mortality. We then built multivariable models, choosing covariates on the basis of biological plausibility, prior literature, and the study objective. Because 28-day mortality events were limited, we kept the number of covariates in each model small to reduce overfitting.

We grouped the primary source of infection as respiratory, urinary or hepatobiliary, or other, with the other sources category used as the reference. Urinary and hepatobiliary sources were combined a priori into a single category. We made this grouping for confounding adjustment, not to estimate the effect of either source on its own or to suggest that the two are clinically the same. Both carry a relatively favorable prognosis in bloodstream infection and sepsis, with lower mortality than respiratory, intra-abdominal, and other infection sources [16,19,32]. In the largest of these cohorts, urinary and biliary sources showed almost identical reductions in mortality, with adjusted odds ratios (aORs) of 0.49 and 0.53, respectively [32]. We retained this combined form in the multivariable models and report outcomes for urinary and hepatobiliary infections separately, as a descriptive analysis, in Appendix B, Table A1.

To evaluate the independent association between Gram status and 28-day mortality after accounting for comorbidity burden, illness severity, infection source, and inappropriate empirical antibiotic therapy, multivariable logistic regression models were constructed. Model 1 included Gram status, age, the Charlson Comorbidity Index, infection source, and the SOFA score. Model 2 additionally incorporated inappropriate empirical antibiotic therapy. As a sensitivity analysis, Model 3 replaced the SOFA score with the presence of septic shock as an alternative, clinically recognizable marker of illness severity. The SOFA score and septic shock were not entered into the same model to avoid multicollinearity between these measures of severity. Multicollinearity among covariates in all multivariable models was assessed using variance inflation factors. We summarized the adjusted estimates and 95% CIs from the three multivariable models as a forest plot.

Missing data were assessed for all study variables. The covariates required for the multivariable models were complete for every patient, so neither case exclusion nor imputation was required for the regression analyses. Variables with missing values were limited to descriptive laboratory measurements, including arterial blood gas and lactate values, which were summarized using available data and were not entered directly as covariates in the multivariable models. The SOFA score, which incorporates the PaO_2_/FiO_2_ ratio, remained complete after application of the predefined respiratory component adjudication rule. The extent of missingness for descriptive variables is reported in the relevant table footnotes.

Two post hoc exploratory analyses were conducted. First, to examine whether inappropriate empirical antibiotic therapy was associated with 28-day mortality, we constructed multivariable logistic regression models that excluded Gram status and adjusted for age, the Charlson Comorbidity Index, infection source, and illness severity, with illness severity modeled using either the SOFA score or the presence of septic shock. Second, we modeled inappropriate empirical antibiotic therapy as the outcome and Gram status as the main exposure, using the same covariate set, to examine whether GPB itself was linked to a higher likelihood of inappropriate therapy. These post hoc exploratory analyses were intended to describe the relationship among Gram status, inappropriate empirical antibiotic therapy, and 28-day mortality, and they were not intended to estimate causal mediation or indirect effects.

The results are reported as odds ratios (ORs) or aORs with 95% confidence intervals (CIs). No formal adjustment for multiple comparisons was applied because secondary and post hoc exploratory analyses were considered hypothesis-generating. As an additional descriptive supplementary analysis, clinical outcomes, source control intervention, and empirical antibiotic appropriateness were summarized according to the four infection source categories used in the multivariable models (respiratory tract, urinary tract, hepatobiliary tract, and other sources). No formal hypothesis testing was performed for this supplement. The comparison of individual infection sources between groups was likewise descriptive, and the corresponding *p* values were not adjusted for multiple comparisons; these per-source *p* values should therefore be regarded as exploratory rather than confirmatory. The infection source variable used in the multivariable regression models was pre-specified as three categories on biological and prior-literature grounds and was not derived from these per-source comparisons.

Analyses were performed using SPSS version 30.0 (IBM Corp., Armonk, NY, USA) for descriptive statistics and group comparisons and R software version 4.4.3 (R Foundation for Statistical Computing, Vienna, Austria) for regression analyses. A two-sided *p* value < 0.05 indicated statistical significance.

## 3. Results

**Figure 1:**
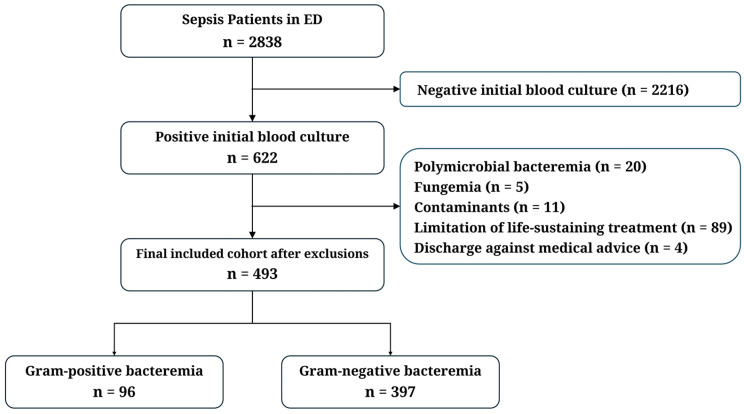
A flowchart of the study population. ED, emergency department.

To compare the clinical characteristics and outcomes of GPB and GNB in patients with sepsis, we began with 2838 adult patients with sepsis presenting to the ED of Keimyung University Dongsan Hospital from December 2021 to August 2024. Of these, 2216 had negative blood cultures and were excluded. Additional exclusions included patients with polymicrobial bloodstream infections (*n* = 20); fungal bloodstream infections (*n* = 5); blood culture isolates deemed contaminants and not treated with antimicrobial therapy (*n* = 11); limitation of life-sustaining treatment, including vasopressors, mechanical ventilation, or renal replacement therapy (*n* = 89); and discharge against medical advice during treatment (*n* = 4). After these exclusions, 493 patients remained, of whom 96 had GPB and 397 had GNB (Figure 1).

### 3.1. Baseline Characteristics

The two groups were comparable in terms of baseline demographics, including age, sex, BMI, and place of acquisition (Table 1). Functional status differed, however, with dependent patients more common in the GPB group (34.4% vs. 22.2%, *p* = 0.037). Regarding comorbidities, ESRD requiring hemodialysis (13.5% vs. 4.0%, *p* < 0.001) and the presence of a central venous catheter (15.6% vs. 7.6%, *p* = 0.014) were more prevalent in the GPB group, whereas the remaining comorbidities were comparable between the groups (Table 1). The prevalence of MDR bacteria was comparable between the groups (42.7% vs. 34.5%, *p* = 0.133), as were Charlson Comorbidity Index scores (5.0 [3.0–6.0] vs. 5.0 [3.0–6.0], *p* = 0.674). At presentation, the two groups did not differ in mean arterial pressure, heart rate, respiratory rate, or body temperature (Table 1). Similarly, the severity of illness parameters did not show any significant difference between the groups as reflected by septic shock (34.4% vs. 41.1%, *p* = 0.230), the SOFA score (4.5 [3.0–7.0] vs. 5.0 [3.0–8.0], *p* = 0.115), and the APACHE II score (18.0 [12.0–21.0] vs. 18.0 [13.0–23.0], *p* = 0.345).

### 3.2. Infection Sources and Causative Pathogens

**Figure 2:**
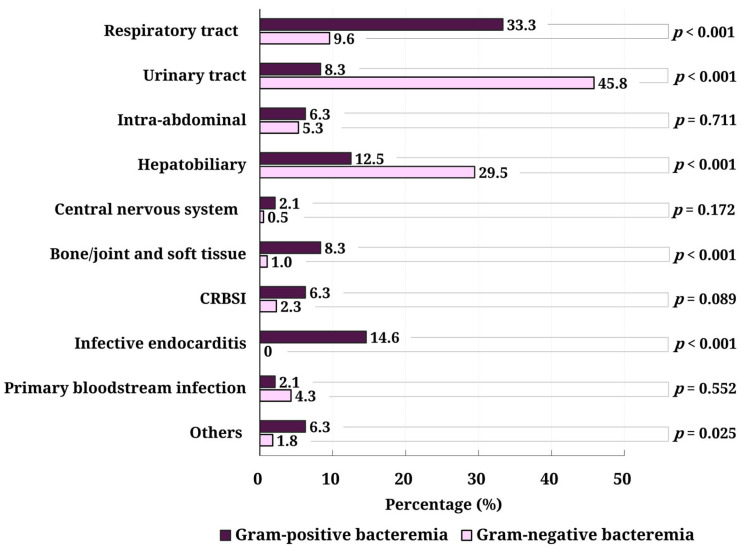
Distribution of infection sources in GPB and GNB. Values represent the percentage of patients with each infection source within each bacteremia group. The *p* values indicate comparisons between the two groups. These *p* values are descriptive and were not adjusted for multiple comparisons. CRBSI, catheter-related bloodstream infection; GPB, Gram-positive bacteremia; GNB, Gram-negative bacteremia.

In descriptive, unadjusted source-specific comparisons, GPB was more frequently associated with respiratory tract infections (33.3% vs. 9.6%, *p* < 0.001); bone, joint, and soft tissue infections (8.3% vs. 1.0%, *p* < 0.001); and infective endocarditis (14.6% vs. 0%, *p* < 0.001). GNB, in contrast, was more frequently associated with hepatobiliary infections (29.5% vs. 12.5%, *p* < 0.001) and urinary tract infections (45.8% vs. 8.3%, *p* < 0.001). Other infection sources were also more frequent in GPB, although this difference was small and based on few patients (6.3% vs. 1.8%, *p* = 0.025). Intra-abdominal infections, central nervous system infections, catheter-related bloodstream infections, and primary bloodstream infections did not differ clearly between the groups. Because the source-specific *p* values were not adjusted for multiple comparisons, these findings were interpreted descriptively, with emphasis on the overall source–distribution pattern rather than confirmatory inference for each individual source category (Figure 2).

**Figure 3:**
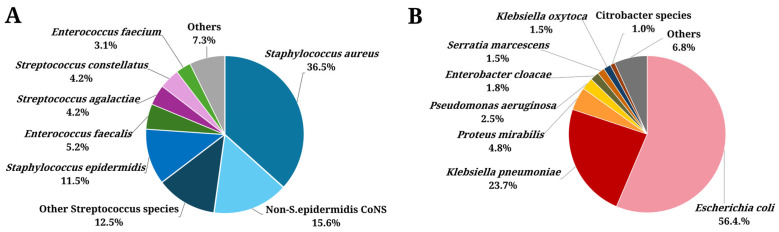
Distribution of causative bacterial species in GPB (**A**) and GNB (**B**). CoNS, coagulase-negative staphylococci; GPB, Gram-positive bacteremia; GNB, Gram-negative bacteremia.

In the GPB group, *Staphylococcus aureus* was the organism isolated most often (36.8%), followed by coagulase-negative staphylococci other than *Staphylococcus epidermidis* (15.6%), *Streptococcus* species other than *S. agalactiae* and *S. constellatus* (12.5%), *Staphylococcus epidermidis* (11.5%), *Enterococcus faecalis* (5.2%), *Streptococcus agalactiae* (4.2%), *Streptococcus constellatus* (4.2%), and *Enterococcus faecium* (3.1%) (Figure 3A). In the GNB group, *Escherichia coli* was the predominant pathogen (56.4%). Other isolates included *Klebsiella pneumoniae* (23.7%), *Proteus mirabilis* (4.8%), *Pseudomonas aeruginosa* (2.5%), and *Enterobacter cloacae* (1.8%) (Figure 3B).

### 3.3. Laboratory and Arterial Blood Gas Parameters

At presentation, the GNB group showed higher lactate levels and lower bicarbonate levels than the GPB group (lactate, 2.7 [1.8–4.6] vs. 1.9 [1.3–3.6] mmol/L, *p* = 0.002; bicarbonate, 20.6 [16.9–24.3] vs. 22.4 [18.7–26.8] mmol/L, *p* = 0.007). Procalcitonin was also higher in GNB (16.3 [3.3–49.7] vs. 4.6 [0.7–18.1] μg/L, *p* < 0.001), while platelet counts were lower (128.0 [80.0–179.0] vs. 161.0 [105.8–222.8] × 10^3^/mm^3^, *p* = 0.001). The remaining ABG and laboratory parameters, including pH, PCO_2_, P/F ratio, WBC count, NLR, hemoglobin, BUN, creatinine, sodium, albumin, and CRP, were broadly similar between the groups (Table 2). Missing values were confined to laboratory measurements and were infrequent, affecting procalcitonin in 17 patients (3.4%) and arterial blood gas and lactate measurements in four patients (0.8%).

### 3.4. Treatment Characteristics and Organ Support

The GNB group received higher crystalloid and total fluid volumes than the GPB group at both 6 and 24 h (Table 3). The time from ED arrival to first antibiotic administration was similar between the groups (2.4 [1.2–4.2] vs. 2.3 [1.4–3.7] hours, *p* = 0.758), whereas inappropriate empirical antibiotic therapy was substantially more frequent in the GPB group than in the GNB group (37.5% vs. 12.8%, *p* < 0.001). Inter-rater agreement for empirical antibiotic appropriateness before consensus was almost perfect (Cohen’s κ = 0.90; 95% CI, 0.85–0.95). Mechanical ventilation within 24 h (26.0% vs. 10.3%, *p* < 0.001) and renal replacement therapy within 24 h (11.5% vs. 5.3%, *p* = 0.028) were more frequently required in the GPB group, whereas source control interventions were more common in the GNB group (44.8% vs. 25.0%, *p* < 0.001). Vasopressor use, use of two or more vasopressors, glucocorticoid therapy for septic shock, and time to source control did not differ between the groups (Table 3).

### 3.5. Primary and Secondary Outcomes

The GPB group had a significantly higher 28-day mortality, the primary outcome, than the GNB group (26.0% vs. 9.3%, *p* < 0.001). The GPB group also showed higher 90-day mortality (34.4% vs. 11.8%, *p* < 0.001), in-hospital mortality (26.0% vs. 10.1%, *p* < 0.001), and ICU admission (36.5% vs. 24.7%, *p* = 0.020). Length of hospital stay was also longer in the GPB group than in the GNB group (20.5 [11.8–31.0] vs. 14.0 [9.0–20.0] days, *p* < 0.001) (Table 4).

### 3.6. Clinical Factors Associated with 28-Day Mortality

In the univariable analysis, GPB was associated with increased 28-day mortality (OR 3.43; 95% CI 1.93–6.03). A higher Charlson Comorbidity Index, a higher SOFA score, septic shock, and inappropriate empirical antibiotic therapy were also associated with increased 28-day mortality (Table 5). In contrast, the combined urinary tract or hepatobiliary source category was associated with lower 28-day mortality, whereas respiratory tract infection was not significantly associated with 28-day mortality (Table 5). Consistent with this, a descriptive analysis by infection source showed lower 28-day and 90-day mortality and more frequent source control in urinary or hepatobiliary infections than in respiratory or other sources (Appendix B, Table A1).

**Figure 4:**
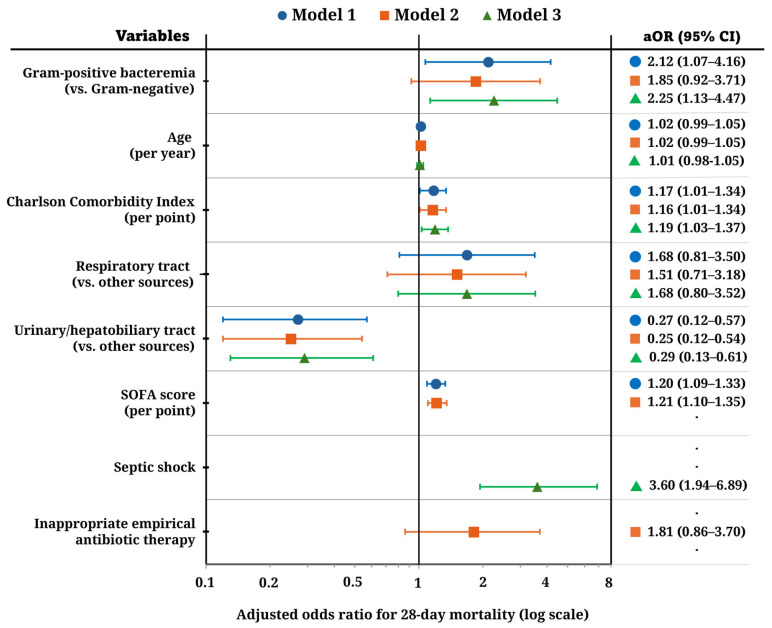
Forest plot of adjusted odds ratios for 28-day mortality in the multivariable logistic regression models. This forest plot presents the adjusted odds ratios (aORs) and 95% confidence intervals (CIs) for the multivariable logistic regression models shown in Table 5. The vertical reference line denotes an aOR of 1.0. Model 1: adjusted for GPB, age, the Charlson Comorbidity Index, source of infection, and the SOFA score. Model 2: Model 1 plus inappropriate empirical antibiotic therapy. Model 3: a sensitivity model based on Model 1, with the SOFA score replaced by septic shock. Across the adjusted models, a higher Charlson Comorbidity Index, greater illness severity (represented by the SOFA score in Models 1 and 2 and by septic shock in Model 3), and a urinary or hepatobiliary infection source remained independently associated with 28-day mortality. GPB remained independently associated with 28-day mortality in Models 1 and 3, whereas this association was attenuated and was no longer statistically significant after adjustment for inappropriate empirical antibiotic therapy in Model 2. aOR, adjusted odds ratio; CI, confidence interval; GPB, Gram-positive bacteremia; SOFA, Sequential Organ Failure Assessment.

In multivariable Model 1, which adjusted for age, the Charlson Comorbidity Index, infection source, and the SOFA score, GPB showed an independent association with 28-day mortality (aOR, 2.12; 95% CI, 1.07–4.16) (Table 5). However, after inappropriate empirical antibiotic therapy was added in Model 2, the association between GPB and 28-day mortality was attenuated and no longer statistically significant (aOR, 1.85; 95% CI, 0.92–3.71). Inappropriate empirical antibiotic therapy was also not independently associated with 28-day mortality in this model (aOR, 1.81; 95% CI, 0.86–3.70). In Model 3, which replaced the SOFA score with septic shock, GPB was independently associated with mortality (aOR, 2.25; 95% CI, 1.13–4.47). The Charlson Comorbidity Index, illness severity (represented by the SOFA score in Models 1 and 2 and by septic shock in Model 3), and the urinary or hepatobiliary source retained consistent associations across models (Figure 4).

In post hoc exploratory models excluding GPB, inappropriate empirical antibiotic therapy was associated with increased 28-day mortality after adjustment for age, the Charlson Comorbidity Index, infection source, and illness severity. This association was observed in both the SOFA-based model (aOR, 2.10; 95% CI, 1.05–4.23) and the septic shock-based model (aOR, 2.22; 95% CI, 1.08–4.53) (Appendix B, Table A2). In a separate post hoc exploratory analysis with inappropriate empirical antibiotic therapy as the outcome, GPB was associated with approximately four-fold higher odds of inappropriate therapy compared with GNB in the unadjusted model (OR, 4.07; 95% CI, 2.45–6.76), the SOFA-based adjusted model (aOR, 4.07; 95% CI, 2.19–7.54), and the septic shock-based adjusted model (aOR, 4.03; 95% CI, 2.17–7.50) (Appendix B, Table A3). Across these models, GPB and inappropriate empirical antibiotic therapy were each associated with 28-day mortality when the other variable was not included. In the model including both variables, neither GPB nor inappropriate empirical antibiotic therapy reached statistical significance.

## 4. Discussion

Our principal finding was that GPB was associated with worse observed clinical outcomes than GNB in ED patients with sepsis, including higher 28-day, 90-day, and in-hospital mortality; a higher ICU admission rate; and longer length of hospital stay. In multivariable analysis, GPB was associated with increased 28-day mortality after adjustment for comorbidity burden, illness severity, and infection source. The association weakened, however, once we also adjusted for inappropriate empirical antibiotic therapy. In exploratory analyses, inappropriate empirical antibiotic therapy was independently associated with 28-day mortality when GPB was excluded, and GPB was associated with an approximately four-fold higher likelihood of receiving inappropriate empirical antibiotic therapy. These patterns suggest that the mortality signal associated with GPB may overlap with inappropriate empirical therapy, although this observation should be interpreted as hypothesis-generating rather than evidence of a causal or mediating effect. Overall, GPB may be better interpreted as a marker of a clinically vulnerable and management-challenging profile rather than as a standalone microbiologic determinant.

Few prior studies have directly compared outcomes between GPB and GNB specifically in patients with bacteremic sepsis. Søgaard et al. reported higher early mortality in community-acquired GPB than in GNB, but later mortality endpoints, including 28-day mortality, were not evaluated [16]. Conversely, an earlier classification study of microbiologically confirmed infections in critically ill patients suggested higher mortality with GNB and highlighted the prognostic importance of both causative organism and infection site [19]. More recent sepsis studies, although not restricted to patients with bacteremia, have also reported inconsistent findings. A large MIMIC-IV analysis reported worse 28-day outcomes in Gram-positive infections, whereas a systematic review and meta-analysis of 45 studies found no significant survival difference between Gram-positive and Gram-negative infections [17,18]. These differences may reflect variation in patient selection, the inclusion of non-bacteremic infections, infection source distribution, and adjustment for illness severity and comorbidity burden. We restricted the cohort to patients with monomicrobial, blood culture-confirmed bacteremic sepsis and adjusted for comorbidity burden, illness severity, infection source, and empirical antibiotic appropriateness. This approach allowed a focused assessment of whether observed outcome differences between GPB and GNB reflect Gram status itself or accompanying patient-, source-, and treatment-related factors in this high-risk subgroup.

The anatomical source of infection is a major prognostic factor in bacteremia and may partly explain observed outcome differences between GPB and GNB. Large cohort studies have consistently shown lower mortality in bacteremia from urinary and hepatobiliary sources than in other infection sources [19,32]. This more favorable prognosis is clinically plausible because urinary and hepatobiliary infections can often be recognized early through clinical findings, urinalysis, hepatic biochemistry, ultrasonography, and computed tomography, which facilitates timely diagnosis and source-directed management. Moreover, even when imaging or laboratory evaluation fails to localize the focus, commonly used empirical regimens for suspected sepsis generally provide reliable coverage of Gram-negative enteric organisms. Because urinary and hepatobiliary infections are predominantly Gram-negative, they may therefore be more likely to receive adequate initial coverage. In urinary tract infections specifically, many renally eliminated antibiotics often achieve high urinary concentrations, which may further enhance antimicrobial activity at the source [33,34]. Importantly, the prognostic effect of infection source appears to persist even within the same causative organism. Studies of *E. coli*, *S. aureus*, and *P. aeruginosa* bacteremia have shown that, within each organism, urinary-source bacteremia is associated with lower mortality than bacteremia arising from non-urinary sources [35,36,37,38,39,40,41]. The relatively favorable prognosis of hepatobiliary-source bacteremia may similarly reflect the feasibility of procedural source control. Percutaneous and endoscopic drainage are the cornerstone of the management of pyogenic liver abscess and acute cholangitis, and timely source control combined with appropriate antibiotic therapy has been associated with improved survival in bacteremic sepsis [42,43,44]. In our cohort, urinary and hepatobiliary source was consistently associated with lower 28-day mortality across all adjusted models (aOR range, 0.25–0.29), supporting the interpretation that differences in infection source distribution may have contributed to the observed outcome differences between GPB and GNB. Consistent with this, a descriptive source category analysis showed that urinary or hepatobiliary infections had lower mortality and more frequent source control than respiratory or other sources (Appendix B, Table A1). These findings highlight suspected infection source as a clinically actionable domain for early ED assessment, empirical antibiotic selection, and source control planning in bacteremic sepsis.

A notable treatment-related finding was the substantially higher frequency of inappropriate empirical antibiotic therapy in GPB than in GNB (37.5% vs. 12.8%). Although inappropriate empirical antibiotic therapy was associated with increased 28-day mortality in univariable analysis, it did not retain an independent association with mortality when modeled together with GPB, and the association between GPB and mortality was also attenuated. However, in post hoc exploratory models excluding Gram status, inappropriate empirical antibiotic therapy was associated with increased 28-day mortality after adjustment for age, comorbidity burden, infection source, and illness severity (Appendix B, Table A2). The association between GPB and inappropriate empirical therapy also persisted after adjustment, with GPB showing approximately four-fold higher odds of inappropriate empirical therapy than GNB (Appendix B, Table A3). These findings suggest that inappropriate empirical therapy is closely linked to the higher-risk profile observed in GPB, but they should not be interpreted as evidence that inappropriate empirical therapy mediated the mortality difference. A more clinically tractable question is why inappropriate empirical therapy was so much more common in GPB. Current sepsis guidelines recommend empirical coverage against methicillin-resistant *Staphylococcus aureus* (MRSA) for patients at high risk of MRSA infection [1,27]. Several GPB-associated clinical features identified in our cohort, including hemodialysis dependence and central venous catheter use, overlap substantially with the risk factors listed in these guidelines. Nevertheless, a high proportion of GPB patients received inappropriate empirical antibiotic therapy, suggesting a modifiable diagnostic–treatment gap in early ED recognition and empirical antibiotic selection before organism identification. This gap may reflect the practical difficulty of operationalizing guideline-based, risk-adapted Gram-positive coverage in the ED [1,45,46]. The association between inappropriate empirical therapy and mortality in our univariable and post hoc exploratory analyses is consistent with prior evidence linking inadequate empirical therapy to worse outcomes in bacteremic sepsis [26,31,47]. Future studies should evaluate whether systematic reassessment of clinical features suggestive of GPB and the suspected infection source during the early ED course can improve empirical antibiotic appropriateness and whether such improvement translates into better clinical outcomes. Rapid diagnostic approaches may further support this strategy, but their clinical value in this setting requires prospective evaluation.

The worse outcomes in GPB are unlikely to be explained by Gram status alone. They appear to reflect a clinically vulnerable and management-challenging profile in which functional status, healthcare exposure, infection source distribution, early organ-support needs, and empirical antibiotic appropriateness converge in early sepsis care. Importantly, this vulnerability was not reflected in a higher Charlson Comorbidity Index, which did not differ between groups (Table 1). Instead, it was expressed through functional dependence (34.4% vs. 22.2%), hemodialysis dependence (13.5% vs. 4.0%), and central venous catheter use (15.6% vs. 7.6%), suggesting a clinically vulnerable and healthcare-exposed profile that may not be fully captured by conventional comorbidity scoring. Functional dependence may indicate reduced physiologic reserve and care dependence, whereas hemodialysis dependence and central venous catheter use more directly reflect healthcare exposure and device-related risk for Gram-positive bloodstream infection. Despite comparable baseline illness severity scores and a numerically lower proportion of septic shock at presentation, GPB patients more frequently required mechanical ventilation within 24 h of ED arrival (26.0% vs. 10.3%) and renal replacement therapy (11.5% vs. 5.3%), indicating greater need for early organ support. Source control was also performed less frequently in GPB than in GNB (25.0% vs. 44.8%), which may partly reflect differences in infection source distribution, including a higher frequency of pneumonia and infective endocarditis in the GPB group. Initial lactate levels were lower in GPB patients than in GNB patients (median, 1.9 vs. 2.7 mmol/L), suggesting that the worse outcomes were not attributable to greater early lactate elevation alone. The higher 90-day mortality in GPB (34.4% vs. 11.8%) suggests that the prognostic disadvantage associated with GPB extended beyond the acute phase, although the mechanisms underlying this longer-term difference could not be determined in this study. Collectively, these observations support the interpretation of GPB as a marker of a clinically vulnerable and management-challenging profile, rather than an isolated microbiologic finding.

From a clinical perspective, our study does not offer a validated tool for predicting GPB, but it highlights two practical gaps in ED care: the early recognition of patients with a GPB-associated high-risk profile and the adequacy of empirical antibiotic coverage. Patients ultimately found to have GPB had worse observed outcomes and substantially more frequent inappropriate empirical therapy. However, the independent contributions of Gram status and inappropriate empirical antibiotic therapy could not be clearly separated in our data. Because our observational design cannot determine which strategy would best address this gap, we do not propose a specific clinical algorithm or any rule for routine empirical anti-MRSA or broad Gram-positive escalation. Rather, our findings support the need for systematic reassessment of clinical features suggestive of GPB and suspected infection source during the early ED course to improve the appropriateness of empirical antibiotic therapy. In our cohort, GPB was associated with functional dependence, hemodialysis dependence, central venous catheter use, recent healthcare exposure, and infection sources such as skin and soft tissue infection, bone and joint infection, CRBSI, pneumonia, and infective endocarditis. These features, together with established considerations such as prior microbiologic results, including MRSA or vancomycin-resistant *Enterococcus* (VRE) infection or colonization, local resistance patterns, and illness severity, may help guide the selection or reassessment of empirical antimicrobial therapy in the ED. Such reassessment may help clinicians decide whether to add empirical Gram-positive coverage based on individual risk, rather than applying broad coverage by default.

Making these reassessment decisions reliably is difficult. The diagnostic–treatment gap described above highlights a point at which structured support may be useful. Decisions about anti-MRSA or broader Gram-positive coverage continue to depend heavily on clinician judgment before organism identification and susceptibility results are available. The cues that may indicate GPB risk, including functional dependence, hemodialysis dependence, central venous catheter use, recent healthcare exposure, the suspected source, prior microbiology, local resistance patterns, and early organ-support needs, are dispersed across the clinical record and may be missed during time-sensitive prescribing. Clinician-supervised, AI-enabled decision support could integrate these cues and prompt earlier reassessment of Gram-positive coverage. An ICU-based machine-learning model predicted empirical antibiotic appropriateness from routine electronic health record data approximately 48 h before complete antibiogram results, with an AUROC of 77.3% in validation [48]. Related decision-support approaches have also been evaluated in ED settings, including host-response classifiers for bacterial infection and 30-day mortality prediction and sepsis alert systems associated with lower mortality in ED cohorts [49]. Such tools should serve as stewardship aids rather than as substitutes for clinical judgment or as justification for indiscriminate broad-spectrum escalation [49,50]. Their potential value may be greatest across the early ED course and ED-to-ICU transition, where empirical regimens are revisited as microbiologic data return and organ dysfunction evolves [50]. These approaches were neither derived nor tested in the present study. Prospective multicenter validation, local calibration, workflow testing, and safeguards for privacy, accountability, and antimicrobial stewardship would be needed before routine use [49,50].

This study has several limitations. As a single-center retrospective study conducted at a tertiary referral hospital, the findings are subject to unmeasured confounding, selection bias, local practice effects, and limited external validity, and they are best used to generate hypotheses rather than to change current practice. Eligible patients were identified by a code-based electronic screen and confirmed by a manual chart review of every screened case, so presentations not captured by the screening codes could still have been missed. We excluded patients with documented limitation of life-sustaining treatment, which reduced the risk of attributing treatment-limitation deaths to infection severity or antibiotic appropriateness but may have removed the most severely ill patients. Our mortality estimates therefore apply primarily to patients managed with full active treatment and may underestimate the absolute mortality of bacteremic ED sepsis, and whether the association between Gram status and outcome differs under treatment limitation remains uncertain. The small GPB group and few mortality events limited statistical power and widened the confidence intervals in the adjusted models. The attenuation of the GPB-mortality association after adjustment for inappropriate empirical antibiotic therapy may reflect overlapping prognostic pathways, but should not be interpreted as mediation, because formal mediation analysis would have been unreliable given the few events and potential residual confounding. These post hoc, non-prespecified analyses are hypothesis-generating, and the clinically vulnerable, management-challenging profile was characterized descriptively rather than through formal phenotyping or predictive model validation. Grouping organisms by Gram status also aggregates pathogens that differ in resistance, virulence, and treatability, and although we used this dichotomy because it reflects the information available to guide empirical therapy before species identification, the categories remain biologically coarse, and the analysis was not powered for species-level, resistance phenotype, or pathogen treatment effects. Infection source distribution also differed between groups, and although we adjusted for prespecified source categories and provided source-stratified analyses, residual source-related confounding likely remains because respiratory, endovascular, urinary, and hepatobiliary infections differ in ease of recognition, empirical coverage, source-control feasibility, and baseline prognosis. The definition of inappropriate empirical antibiotic therapy combined in vitro susceptibility with source- and pathogen-specific guideline concordance, and although inter-rater agreement was high, some interpretation-dependent misclassification remains possible. Our analysis focused on empirical antibiotic treatment within the first 24 h and did not model exact timing to effective coverage or subsequent antibiotic changes, limiting interpretation of treatment pathways. Finally, the ED-based risk stratification and AI-enabled decision-support approaches discussed here were neither derived nor tested in this study, and prospective multicenter studies are needed to determine whether systematic early-ED reassessment of features suggestive of GPB and of the suspected infection source improves empirical antibiotic appropriateness and clinical outcomes without unnecessary escalation.

## 5. Conclusions

In bacteremic sepsis, GPB was associated with higher observed 28-day and 90-day mortality, more frequent ICU admission, longer hospitalization, and substantially more frequent inappropriate empirical antibiotic therapy than GNB. GPB should be interpreted less as a standalone microbiologic determinant of mortality and more as a marker of a clinically vulnerable and management-challenging profile. Our findings point to a modifiable diagnostic–treatment gap in early ED recognition and empirical antibiotic selection. Prospective multicenter studies are warranted to test whether systematic reassessment of clinical features suggestive of GPB and the suspected infection source across the early ED course and ED-to-ICU transition improves empirical antibiotic appropriateness and ultimately clinical outcomes. Clinician-supervised decision support tools may support this reassessment.

## Figures and Tables

**Table 1:** The baseline characteristics of patients with GPB and GNB.

Variables	GPB	GNB	*p* Value
Number of patients (*n*)	96	397	
Age (years)	76.0 (66.0–81.3)	75.0 (67.0–83.0)	0.585
Sex, male	63 (65.6)	223 (56.2)	0.092
BMI (kg/m^2^)	22.6 (20.0–25.4)	23.2 (20.8–25.9)	0.176
Functional status			0.037
Independent	58 (60.4)	276 (69.5)	
Partially dependent	5 (5.2)	33 (8.3)	
Dependent	33 (34.4)	88 (22.2)	
Place of acquisition			0.217
Community	68 (70.8)	306 (77.1)	
Nosocomial	25 (26.0)	72 (18.1)	
Nursing home	3 (3.1)	19 (4.8)	
Comorbidities			
HTN	56 (58.3)	203 (51.1)	0.205
DM	35 (36.5)	174 (43.8)	0.190
Heart failure	5 (5.2)	10 (2.5)	0.184
Chronic lung disease	6 (6.3)	20 (5.0)	0.633
Non-dialysis CKD	6 (6.3)	28 (7.1)	0.781
ESRD on hemodialysis	13 (13.5)	16 (4.0)	<0.001
Chronic liver disease	8 (8.3)	47 (11.8)	0.328
Transplantation	1 (1.0)	9 (2.3)	0.695
Solid tumor	11 (11.5)	69 (17.4)	0.158
Hematologic malignancy *	2 (2.1)	12 (3.0)	0.999
Severe neutropenia (ANC < 500 cells/µL)	2 (2.1)	9 (2.3)	0.999
Recent chemotherapy †	6 (6.3)	34 (8.6)	0.456
Systemic corticosteroid therapy §	4 (4.2)	9 (2.3)	0.292
Central venous catheter	15 (15.6)	30 (7.6)	0.014
Charlson Comorbidity Index	5.0 (3.0–6.0)	5.0 (3.0–6.0)	0.674
Multidrug-resistant bacteria **	41 (42.7)	137 (34.5)	0.133
Septic shock	33 (34.4)	163 (41.1)	0.230
SOFA score	4.5 (3.0–7.0)	5.0 (3.0–8.0)	0.115
APACHE II score	18.0 (12.0–21.0)	18.0 (13.0–23.0)	0.345
Initial vital signs			
Mean arterial pressure (mmHg)	81.0 (67.0–99.0)	80.0 (67.0–97.0)	0.614
Heart rate (beats/min)	97.0 (79.5–112.0)	99.5 (82.0–115.0)	0.670
Respiratory rate (breaths/min)	20.0 (20.0–21.0)	20.0 (20.0–20.0)	0.114
Temperature (°C)	37.6 (36.7–38.5)	37.8 (36.8–38.5)	0.427

Values are presented as medians (interquartile ranges) or numbers (%). * Hematologic malignancy was defined as leukemia, myelodysplastic syndrome, myeloproliferative neoplasm, lymphoma, multiple myeloma, or other plasma-cell or hematolymphoid malignancy, restricted to patients receiving disease-directed treatment at ED presentation; remote disease in remission without current disease-directed treatment was not classified as having hematologic malignancy. † Recent chemotherapy was defined as cytotoxic chemotherapy or disease-directed systemic anticancer therapy within the past 3 months. § Systemic corticosteroid therapy was defined as treatment with >20 mg of prednisolone per day or an equivalent dose for at least 2 weeks within the past month. ** Multidrug-resistant bacteria were defined as isolates resistant to at least one agent in three or more antimicrobial categories. GPB, Gram-positive bacteremia; GNB, Gram-negative bacteremia; ESRD, end-stage renal disease; ANC, absolute neutrophil count; SOFA, Sequential Organ Failure Assessment; APACHE II, Acute Physiology and Chronic Health Evaluation II.

**Table 2:** Initial laboratory and arterial blood gas parameters in patients with GPB and GNB.

Variables	GPB	GNB	*p* Value
Initial ABG and lactate			
pH	7.4 (7.4–7.5)	7.4 (7.4–7.5)	0.130
PCO_2_ (mmHg)	33.3 (27.9–38.0)	31.6 (26.1–37.7)	0.122
P/F ratio	241.4 (162.2–360.2)	294.1 (174.9–373.3)	0.137
HCO_3_ (mmol/L)	22.4 (18.7–26.8)	20.6 (16.9–24.3)	0.007
Lactate (mmol/L)	1.9 (1.3–3.6)	2.7 (1.8–4.6)	0.002
Initial laboratory parameters			
WBC (×10^3^/mm^3^)	11.9 (8.6–16.6)	10.9 (6.8–16.7)	0.093
NLR	16.1 (10.0–30.1)	19.6 (10.2–30.1)	0.329
Hemoglobin (g/dL)	11.1 (9.4–12.7)	11.2 (9.8–12.8)	0.354
Platelet (×10^3^/mm^3^)	161.0 (105.8–222.8)	128.0 (80.0–179.0)	0.001
BUN (mg/dL)	34.5 (24.0–54.3)	31.0 (20.0–47.0)	0.139
Creatinine (mg/dL)	1.6 (1.0–2.9)	1.5 (1.1–2.7)	0.751
Na (mmol/L)	133.5 (131.0–138.0)	134.0 (130.0–137.0)	0.306
Albumin (g/dL)	3.2 (2.7–3.6)	3.2 (2.8–3.7)	0.094
CRP (mg/dL)	16.7 (9.0–23.7)	14.1 (7.8–22.4)	0.181
Procalcitonin (μg/L)	4.6 (0.7–18.1)	16.3 (3.3–49.7)	<0.001

Values are presented as medians (interquartile ranges). Data were missing for procalcitonin in 17 patients (3.4%) and for initial lactate and arterial blood gas-derived variables, including pH, PaCO_2_, HCO_3_, and the P/F ratio, in 4 patients (0.8%). Descriptive summaries were based on available non-missing data. GPB, Gram-positive bacteremia; GNB, Gram-negative bacteremia; ABG, arterial blood gas; P/F ratio, PaO_2_/FiO_2_ ratio; NLR, neutrophil-to-lymphocyte ratio.

**Table 3:** Treatment, interventions, and organ support in patients with GPB and GNB.

Variables	GPB	GNB	*p* Value
Fluid management			
6 h crystalloid volume (mL)	685.0 (247.5–1257.5)	920.0 (440.0–1580.0)	0.011
6 h total fluid volume * (mL)	810.0 (315.0–1500.5)	1000.0 (480.0–1700.0)	0.016
24 h crystalloid volume (mL)	1530.0 (930.0–2487.5)	2080.0 (1300.0–3060.0)	0.001
24 h total fluid volume * (mL)	2360.0 (1565.0–3340.0)	2800.0 (1920.0–3920.0)	0.010
Antibiotic therapy			
Time to first antibiotic administration (hours)	2.4 (1.2–4.2)	2.3 (1.4–3.7)	0.758
Inappropriate empirical antibiotic therapy †	36 (37.5)	51 (12.8)	<0.001
Organ support therapy			
Mechanical ventilation within 24 h	25 (26.0)	41 (10.3)	<0.001
Renal replacement therapy within 24 h	11 (11.5)	21 (5.3)	0.028
Vasopressor	39 (40.6)	199 (50.1)	0.095
Use of ≥2 vasopressors	18 (18.8)	72 (18.1)	0.889
Glucocorticoid for septic shock	17 (17.7)	67 (16.9)	0.531
Source control intervention	24 (25.0)	178 (44.8)	<0.001
Time to source control intervention (hours)	7.5 (2.8–17.4)	6.5 (4.0–15.0)	0.439

Values are presented as medians (interquartile ranges) or numbers (%). * Total fluid includes crystalloids, colloids, and free water. † Defined as empirical therapy lacking in vitro activity against the cultured organism or discordant with guideline-recommended regimens based on the infection source and expected pathogens. GPB, Gram-positive bacteremia; GNB, Gram-negative bacteremia.

**Table 4:** Primary and secondary outcomes in patients with GPB and GNB.

Variables	GPB	GNB	*p* Value
Primary outcome			
28-day mortality	25 (26.0)	37 (9.3)	<0.001
Secondary outcomes			
90-day mortality	33 (34.4)	47 (11.8)	<0.001
In-hospital mortality	25 (26.0)	40 (10.1)	<0.001
ICU admission	35 (36.5)	98 (24.7)	0.020
Length of hospital stay (days)	20.5 (11.8–31.0)	14.0 (9.0–20.0)	<0.001

Values are presented as medians (interquartile ranges) or numbers (%).

**Table 5:** Univariable and multivariable logistic regression analyses for 28-day mortality.

Variables	Unadjusted	Adjusted (Model 1)	Adjusted (Model 2)	Adjusted (Model 3)
	OR (95% CI)	aOR (95% CI)	aOR (95% CI)	aOR (95% CI)
GPB	3.43 (1.93–6.03)	2.12 (1.07–4.16)	1.85 (0.92–3.71)	2.25 (1.13–4.47)
Age	1.02 (1.00–1.05)	1.02 (0.99–1.05)	1.02 (0.99–1.05)	1.01 (0.98–1.05)
Charlson Comorbidity Index	1.25 (1.12–1.41)	1.17 (1.01–1.34)	1.16 (1.01–1.34)	1.19 (1.03–1.37)
Source of infection				
Other sources *	Reference	Reference	Reference	Reference
Respiratory tract	1.72 (0.87–3.42)	1.68 (0.81–3.50)	1.51 (0.71–3.18)	1.68 (0.80–3.52)
Urinary tract or hepatobiliary †	0.19 (0.09–0.37)	0.27 (0.12–0.57)	0.25 (0.12–0.54)	0.29 (0.13–0.61)
SOFA score	1.21 (1.10–1.32)	1.20 (1.09–1.33)	1.21 (1.10–1.35)	—
Septic shock	3.78 (2.17–6.79)	—	—	3.60 (1.94–6.89)
Inappropriate empirical antibiotics	2.15 (1.15–3.88)	—	1.81 (0.86–3.70)	—

* Other sources include all infection sites not classified as respiratory, urinary, or hepatobiliary infections (e.g., bone/joint and soft tissue, intra-abdominal, central nervous system, infective endocarditis, catheter-related bloodstream infections, and primary bloodstream infections). † The urinary tract or hepatobiliary category included patients with either urinary tract or hepatobiliary infection as the primary source. Em dashes (—) indicate that the variable was not included in the corresponding model. Model 1: Adjusted for GPB, age, the Charlson Comorbidity Index, source of infection, and the SOFA score. Model 2: Adjusted for GPB, age, the Charlson Comorbidity Index, source of infection, the SOFA score, and inappropriate empirical antibiotic therapy. Model 3: Adjusted for GPB, age, the Charlson Comorbidity Index, source of infection, and septic shock. GPB, Gram-positive bacteremia; GNB, Gram-negative bacteremia; SOFA, Sequential Organ Failure Assessment.

## Data Availability

The data presented in this study are available on reasonable request from the corresponding author. The data are not publicly available due to patient privacy considerations.

## Supplementary Materials

The following supporting information can be downloaded at https://www.mdpi.com/article/10.3390/medicina62071353/s1: Table S1: KCD-8 codes used for initial infectious disease screening.

## Abbreviations

The following abbreviations are used in this manuscript:

ABG	Arterial blood gas
aOR	Adjusted odds ratio
APACHE II	Acute Physiology and Chronic Health Evaluation II
BMI	Body mass index
BUN	Blood urea nitrogen
CI	Confidence interval
CKD	Chronic kidney disease
CoNS	Coagulase-negative staphylococci
CRP	C-reactive protein
ED	Emergency department
ESRD	End-stage renal disease
GNB	Gram-negative bacteremia
GPB	Gram-positive bacteremia
HCO_3_	Bicarbonate
ICD-10	International Classification of Diseases, 10th Revision
ICU	Intensive care unit
MRSA	Methicillin-resistant Staphylococcus aureus
MSSA	Methicillin-susceptible Staphylococcus aureus
Na	Sodium
NLR	Neutrophil-to-lymphocyte ratio
OR	Odds ratio
PCO_2_	Partial pressure of carbon dioxide
P/F ratio	PaO_2_/FiO_2_ ratio
SOFA	Sequential Organ Failure Assessment
WBC	White blood cell

## Appendix B

**Table A1:** Descriptive clinical outcomes according to infection source category.

Variables	Respiratory Tract	Hepatobiliary	Urinary Tract	Other Sources *****
Number of patients	70	190	129	104
GPB	32 (45.7)	8 (4.2)	12 (9.3)	44 (42.3)
GNB	38 (54.3)	182 (95.8)	117 (90.7)	60 (57.7)
28-day mortality	23 (32.9)	11 (5.8)	5 (3.9)	23 (22.1)
90-day mortality	30 (42.9)	14 (7.4)	8 (6.2)	28 (26.9)
ICU admission	31 (44.3)	41 (21.6)	16 (12.4)	45 (43.3)
Length of hospital stay (days)	16 (8–31)	14 (10–20)	12 (8–18)	19 (12–29)
Source control intervention	9 (12.9)	52 (27.4)	119 (92.2)	22 (21.2)
Inappropriate empirical antibiotic therapy	24 (34.3)	26 (13.7)	21 (16.3)	16 (15.4)

Values are presented as n, n (%) or median (IQR). Percentages were calculated within each infection source category. * Other sources include all infection sites not classified as respiratory tract, urinary tract, hepatobiliary infections (e.g., bone/joint and soft tissue, intra-abdominal, central nervous system, infective endocarditis, catheter-related bloodstream infections, and primary bloodstream infections). GPB, Gram-positive bacteremia; GNB, Gram-negative bacteremia; ICU, intensive care unit.

**Table A2:** Post hoc exploratory multivariable logistic regression analyses for 28-day mortality excluding Gram status.

Variables	Adjusted (Model 1)		Adjusted (Model 2)	
	aOR (95% CI)	*p* Value	aOR (95% CI)	*p* Value
Age	1.02 (0.99–1.05)	0.273	1.01 (0.98–1.05)	0.387
Charlson Comorbidity Index	1.15 (1.00–1.33)	0.049	1.17 (1.01–1.34)	0.033
Source of infection				
Other sources *	Reference		Reference	
Respiratory tract	1.48 (0.71–3.10)	0.297	1.46 (0.69–3.09)	0.321
Urinary tract or hepatobiliary †	0.20 (0.10–0.42)	<0.001	0.21 (0.10–0.44)	<0.001
SOFA score	1.20 (1.08–1.32)	<0.001	—	—
Septic shock	—	—	3.47 (1.85–6.50)	<0.001
Inappropriate empirical antibiotics	2.10 (1.05–4.23)	0.037	2.22 (1.08–4.53)	0.029

* Other sources include all infection sites not classified as respiratory, urinary, or hepatobiliary infections (e.g., bone/joint and soft tissue, intra-abdominal, central nervous system, infective endocarditis, catheter-related bloodstream infections, and primary bloodstream infections). † The urinary tract or hepatobiliary category included patients with either urinary tract or hepatobiliary infection as the primary source. Em dashes (—) indicate that the variable was not included in the corresponding model. Model 1: Adjusted for age, the Charlson Comorbidity Index, source of infection, the SOFA score, and inappropriate empirical antibiotics. Model 2: Adjusted for age, the Charlson Comorbidity Index, source of infection, septic shock, and inappropriate empirical antibiotics. SOFA, Sequential Organ Failure Assessment.

**Table A3:** Logistic regression analysis of the association between GPB and inappropriate empirical antibiotic therapy.

Variables	Patients, n/N (%)		Unadjusted	Model 1	Model 2
	Appropriate Therapy	Inappropriate Therapy	OR (95% CI)	aOR (95% CI)	aOR (95% CI)
GNB	346/397 (87.2)	51/397 (12.8)	Reference	Reference	Reference
GPB	60/96 (62.5)	36/96 (37.5)	4.07 (2.45–6.76)	4.07 (2.19–7.54)	4.03 (2.17–7.50)

Model 1: Adjusted for age, the Charlson Comorbidity Index, source of infection, and the SOFA score. Model 2: Adjusted for age, the Charlson Comorbidity Index, source of infection, and septic shock. Infection source was categorized as other sources (reference), respiratory tract, and urinary tract or hepatobiliary infection. OR and aORs represent the odds of inappropriate empiric antibiotic therapy in patients with GPB compared with those with GNB. GPB, Gram-positive bacteremia; GNB, Gram-negative bacteremia.
