# Supplementary material for: Clinical Characteristics, Empirical Antibiotic Appropriateness, and Outcomes of Gram-Positive Versus Gram-Negative Bacteremia in Emergency Department Patients with Sepsis
Source: Medicina (Kaunas). 2026 Jul 13;62(7):1353. doi: 10.3390/medicina62071353 (PMC13413519; doi:10.3390/medicina62071353)
Supplement: Supplementary file 1 [file medicina-62-01353-s001.zip › medicina-4378219-supplementary.pdf]

Table S1. KCD-8 codes used for initial infectious disease screening.

Source category	KCD-8*	Diagnosis
Sepsis / Septic shock	A40.0	Streptococcal sepsis, group A
	A40.1	Streptococcal sepsis, group B
	A40.3	Sepsis due to Streptococcus pneumoniae
	A40.8	Other streptococcal sepsis
	A40.9	Streptococcal sepsis, unspecified
	A41	Other sepsis
	A41.0	Sepsis due to Staphylococcus aureus
	A41.1	Sepsis due to other specified staphylococcus
	A41.2	Sepsis due to unspecified staphylococcus
	A41.3	Sepsis due to Haemophilus influenzae
	A41.4	Sepsis due to anaerobes
	A41.5	Sepsis due to other Gram-negative organisms
	A41.8	Other specified sepsis
	A41.9	Sepsis, unspecified organism
	A39.2	Acute meningococcaemia
	A39.4	Meningococcaemia, unspecified
	A32.7	Listerial sepsis
	A42.7	Actinomycotic sepsis
	A26.7	Erysipelothrix sepsis
	R65.2	Severe sepsis
	R57.2	Septic shock
	R65.0	SIRS of infectious origin without organ dysfunction
	R65.1	SIRS of infectious origin with organ dysfunction
	A49.9	Bacterial infection, unspecified
Respiratory tract infection	J13	Pneumonia due to Streptococcus pneumoniae
	J14	Pneumonia due to Haemophilus influenzae
	J15.0	Pneumonia due to Klebsiella pneumoniae
	J15.1	Pneumonia due to Pseudomonas
	J15.2	Pneumonia due to staphylococcus
	J15.3	Pneumonia due to streptococcus, group B
	J15.4	Pneumonia due to other streptococci
	J15.5	Pneumonia due to Escherichia coli
	J15.6	Pneumonia due to other aerobic Gram-negative bacteria
	J15.7	Pneumonia due to Mycoplasma pneumoniae
	J15.8	Other bacterial pneumonia
	J15.9	Bacterial pneumonia, unspecified
	J16.8	Pneumonia due to other specified infectious organisms
	J18.0	Bronchopneumonia, unspecified
	J18.1	Lobar pneumonia, unspecified
	J18.8	Other pneumonia, organism unspecified
	J18.9	Pneumonia, unspecified
	J69.0	Pneumonitis due to food and vomit
	J85.1	Abscess of lung with pneumonia
	J85.2	Abscess of lung without pneumonia
	J85.3	Abscess of mediastinum
	J86.0	Pyothorax with fistula
	J86.9	Pyothorax without fistula
Urinary tract infection	N10	Acute tubulo-interstitial nephritis
	N11.0	Nonobstructive reflux-associated chronic pyelonephritis
	N11.1	Chronic obstructive pyelonephritis
	N11.9	Chronic tubulo-interstitial nephritis, unspecified
	N12	Tubulo-interstitial nephritis, not specified as acute or chronic

	N13.6	Pyonephrosis
	N15.1	Renal and perinephric abscess
	N30.0	Acute cystitis
	N34	Urethritis and urethral syndrome
	N39.0	Urinary tract infection, site not specified
	N41.0	Acute prostatitis
	N41.2	Abscess of prostate
	N45	Orchitis and epididymitis
Intra-abdominal infection	K35.2	Acute appendicitis with generalized peritonitis
	K35.3	Acute appendicitis with localized peritonitis
	K35.8	Acute appendicitis, other and unspecified
	K57.0	Diverticular disease of small intestine with perforation and abscess
	K57.2	Diverticular disease of large intestine with perforation and abscess
	K57.4	Diverticular disease of both small and large intestine with perforation and abscess
	K57.8	Diverticular disease of intestine, part unspecified, with perforation and abscess
	K63.0	Abscess of intestine
	K63.1	Perforation of intestine (nontraumatic)
	K65.0	Acute peritonitis
	K65.8	Other peritonitis
	K65.9	Peritonitis, unspecified
	K61.0	Anal abscess
	K61.1	Rectal abscess
	K61.2	Anorectal abscess
	K61.3	Ischiorectal abscess
	K61.4	Intrasphincteric abscess
Hepatobiliary infection	K80.0	Calculus of gallbladder with acute cholecystitis
	K80.1	Calculus of gallbladder with other cholecystitis
	K80.3	Calculus of bile duct with cholangitis
	K80.4	Calculus of bile duct with cholecystitis
	K81.0	Acute cholecystitis
	K81.8	Other cholecystitis
	K81.9	Cholecystitis, unspecified
	K83.0	Cholangitis
	K83.1	Obstruction of bile duct
	K75.0	Abscess of liver
	K75.1	Phlebitis of portal vein
CNS infection	G00.0	Haemophilus meningitis
	G00.1	Pneumococcal meningitis
	G00.2	Streptococcal meningitis
	G00.3	Staphylococcal meningitis
	G00.8	Other bacterial meningitis
	G00.9	Bacterial meningitis, unspecified
	G01	Meningitis in bacterial diseases classified elsewhere
	G03	Meningitis due to other and unspecified causes
	G04.2	Bacterial meningoencephalitis and meningomyelitis, NEC
	G06.0	Intracranial abscess and granuloma
	G06.1	Intraspinal abscess and granuloma
	G06.2	Extradural and subdural abscess, unspecified
	A39.0	Meningococcal meningitis
Bone, joint and soft tissue infection	M00.0	Staphylococcal arthritis and polyarthritis
	M00.1	Pneumococcal arthritis and polyarthritis
	M00.2	Other streptococcal arthritis and polyarthritis
	M00.8	Arthritis and polyarthritis due to other specified bacterial agents
	M00.9	Pyogenic arthritis, unspecified
	M86.0	Acute haematogenous osteomyelitis

	M86.1	Other acute osteomyelitis
	M86.2	Subacute osteomyelitis
	M86.3	Chronic multifocal osteomyelitis
	M86.4	Chronic osteomyelitis with draining sinus
	M86.5	Other chronic haematogenous osteomyelitis
	M86.6	Other chronic osteomyelitis
	M46.2	Osteomyelitis of vertebra
	M46.3	Infection of intervertebral disc, pyogenic
	L02.0	Cutaneous abscess, furuncle and carbuncle of face
	L02.1	Cutaneous abscess, furuncle and carbuncle of neck
	L02.2	Cutaneous abscess, furuncle and carbuncle of trunk
	L02.3	Cutaneous abscess, furuncle and carbuncle of buttock
	L02.4	Cutaneous abscess, furuncle and carbuncle of limb
	L03.0	Cellulitis of finger and toe
	L03.1	Cellulitis of other parts of limb
	L03.2	Cellulitis of face
	L03.3	Cellulitis of trunk
	L03.8	Cellulitis of other sites
	L03.9	Cellulitis, unspecified
	L08.0	Pyoderma
	L08.9	Local infection of skin and subcutaneous tissue, unspecified
	M72.6	Necrotizing fasciitis
	M60.0	Infective myositis
CRBSI	T80.2	Infections following infusion, transfusion and therapeutic injection
	T82.7	Infection/inflammatory reaction due to other cardiac and vascular devices, implants and grafts
	T85.7	Infection/inflammatory reaction due to other internal prosthetic devices, implants and grafts
Infective endocarditis	I33.0	Acute and subacute infective endocarditis
	I33.9	Acute and subacute endocarditis, unspecified
	I38	Endocarditis, valve unspecified
	I39	Endocarditis and heart valve disorders in diseases classified elsewhere
Others	H44.0	Purulent endophthalmitis
	J01	Acute sinusitis
	J03.0	Streptococcal tonsillitis
	J03.8	Acute tonsillitis due to other specified organisms
	J03.9	Acute tonsillitis, unspecified
	J05.1	Acute epiglottitis
	J36	Peritonsillar abscess
	J39.0	Retropharyngeal and parapharyngeal abscess
	J39.1	Other abscess of pharynx
	J32	Chronic sinusitis
	K05	Gingivitis and periodontal diseases
	K12.2	Cellulitis and abscess of mouth

*KCD-8 is the Korean national disease classification system based on the WHO International Classification of Diseases, Tenth Revision (ICD-10), and has been implemented in Korea since 1 January 2021.

ICD-10, International Classification of Diseases, 10th Revision; KCD-8, Korean Standard Classification of Diseases, 8th Revision; CNS, central nervous system; CRBSI, catheter-related bloodstream infection.
